# Comprehensive review on biotechnological production of hyaluronic acid: status, innovation, market and applications

**DOI:** 10.1080/21655979.2022.2057760

**Published:** 2022-04-18

**Authors:** Ruschoni Ucm, Mera Aem, Zamudio Lhb, Vinod Kumar, Mohammad J. Taherzadeh, Vijay Kumar Garlapati, Anuj Kumar Chandel

**Affiliations:** a Department of Biotechnology, Engineering School of Lorena (EEL), University of São Paulo (USP), Lorena 12602-810, Brazil; b School of Water, Energy and Environment, Cranfield University, Cranfield MK43 0AL, UK; c Swedish Centre for Resource Recovery, University of Borås, S-501 90, Borås, Sweden; d Department of Biotechnology and Bioinformatics, University of Information Technology, Waknaghat 173234, India

**Keywords:** Hyaluronic acid, *streptococcus zooepidemicus*, fermentation, downstream processing, industrial scenario

## Abstract

The growing, existing demand for low-cost and high-quality hyaluronic acid (HA) needs an outlook of different possible production strategies from renewable resources with the reduced possibility of cross-infections. Recently, the possibility of producing HA from harmless microorganisms appeared, which offers the opportunity to make HA more economical, without raw material limitations, and environmentally friendly. HA production is mainly reported with Lancefield *Streptococci A* and *C*, particularly from *S. equi* and *S. zooepidemicus*. Various modes of fermentation such as batch, repeated batch, fed-batch, and continuous culture have been investigated to optimize HA production, particularly from *S. zooepidemicus*, obtaining a HA yield of 2.5 g L^−1^ – 7.0 g L^−1^. Among the different utilized DSP approaches of HA production, recovery with cold ethanol (4°C) and cetylpyridinium chloride is the ideal strategy for lab-scale HA production. On the industrial scale, besides using isopropanol, filtration (0.22 um), ultrafiltration (100 kDa), and activated carbon absorption are employed to obtain HA of low molecular weight and additional ultrafiltration to purify HA of higher MW. Even though mature technologies have already been developed for the industrial production of HA, the projections of increased sales volume and the expansion of application possibilities require new processes to obtain HA with higher productivity, purity, and specific molecular weights. In this review, we have put forth the progress of HA technological research by discussing the microbial biosynthetic aspects, fermentation and downstream strategies, industrial-scale scenarios of HA, and the prospects of HA production to meet the current and ongoing market demands.

## Graphical Abstract

**Figure:**
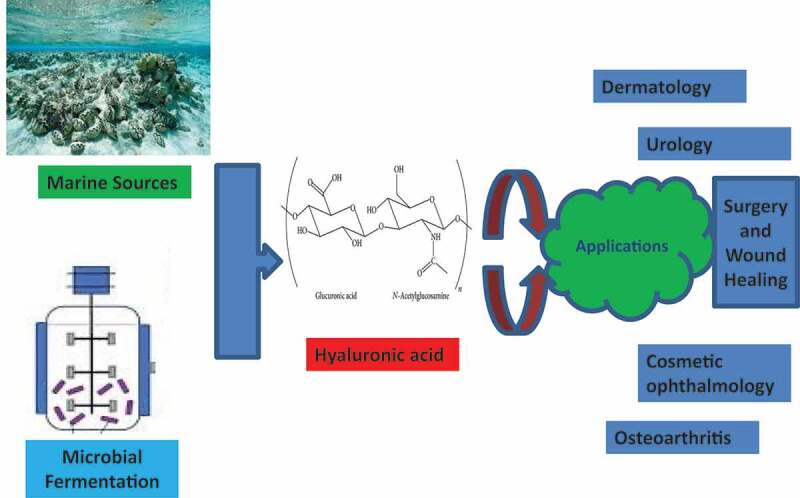

## Highlights

- Hyaluronic acid (HA) is a vital glycosaminoglycan polysaccharide.
- HA occupies a prominent place in medical, pharmaceutical, and polymer segments.
- Biological production is the preferred way for sustainable HA production.
- Fed-batch fermentation mode helps in attaining the higher titers values of HA.
- Discussed the Industrial-scale scenario and future prospects of HA production.

## 1. Introduction

Fermentative production of biomolecules is gaining a constant interest over chemical methods toward an environmentally friendly and sustainable world. There is a growing demand for economically viable processes that avoid harming the environment. Thus, continuous research is needed to produce biodegradable polymers of microbial origin [1]. Hyaluronic acid (HA) is a vital glycosaminoglycan polysaccharide in the vertebrate’s’ extracellular matrix of connective and epithelial tissue (joints synovial fluid, umbilical cord, eye;s vitreous humor, cartilage scaffolds) of vertebrates [2]. HA produced in mammals has an essential biological function. Therefore, the primary source of HA is animal tissues; however, extensive purification protocols are required to prevent toxin contamination [3,4].

**Figure 1.**
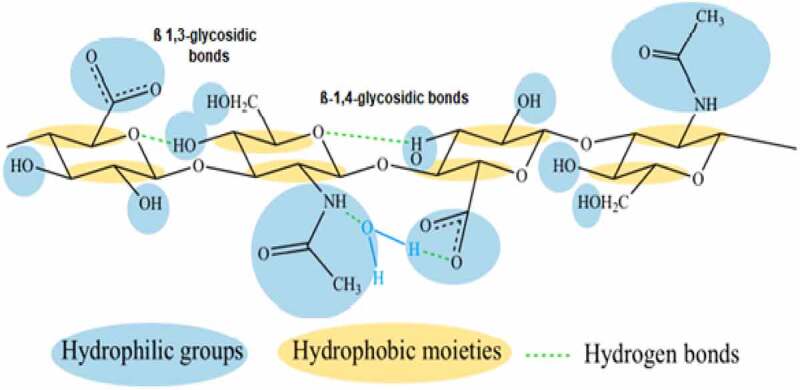
A structure of hyaluronic acid monomers(Blue colour denotes the hydrophilic functional groups, yellow colour depicts the hydrophobic moieties and green-dashed lines shows the presence of hydrogen bonds).

HA is an organic and linear glycosaminoglycan (GAG) biopolymer with the monomeric disaccharide units of D-glucuronic acid (GlcUA) and N-acetyl glucosamine (GlcNAc) connected through alternating glycosidic bonds β-(1→3) and β-(1→4), respectively (Figure 1) (Atkins and Sheehan, 1972). Hyaluronic acid (HA), a linear polymer of natural origin with the building blocks of D-glucuronic acid and N- acetylglucosamine, differs from the other synthetic polymers such as PEG a remarkable biological activity with viscoelasticity, biocompatibility, water-retention, and non-immunogenic properties. It has a molecular weight of approximately 400 Da [5,6]. As a non-immunogenic polymer with a high water-holding capacity, the presence of -COOH and -OH groups makes HA as a promising role in biomaterials sectors toward designing tissue-culture scaffolds and drug-delivery systems [7–10]. Another primary application of HA lies in using cross-linked hydrogels preparation [11,12], which serves as self-assembled aggregates of proteins, drugs, nanoparticles, and gels [13].

HA has a broad range of applications in the medicine and pharmaceutical sectors with 1,000–5,000 USD/Kg, depending on its purity, size, and properties. The chain length of HA dictates the end-use of HA in different physiological and metabolic processes (Dymarska et al., 2016). HA with a 1.0 × 10^6^ Da MW exhibits higher viscosity with moisturizing activities (Schulte et al., 2019). The naturally anionic (-ve charged) HA polymer can bind to more water (expand 1,000 times volume) and form a viscous gel which serves as a lubricant and shock absorber (space filler) for joints and surrounding tissues, respectively [14]. The HA with 1.0 × 10^4^–1.0 × 10^6^ Da MW is used to fabricate various cross-linked products with penultimate usage in chronic wound healing. Further, the HA network also helps connective tissues (vocal folds, cartilage, and vitreous humor) viscoelasticity maintenance [2]. The biocompatibility coupled viscoelasticity properties make HA a probable candidate for different biomedical applications such as drug delivery, tissue engineering, ophthalmic surgeries, and arthritis treatment [15]. HA oligosaccharides with ≤1.0 × 10^4^ Da utilized for the proliferation of fibroblasts and blood vessels with tumor suppression properties [Wanget al.,2020; 15, 50, 72]. The antioxidant capability of HA is attributed to its interaction with the oxygen-derived-free radicals. The inhibition ability of HA in migration and aggregation of macrophages makes HA an inflammation mediator. The high-water absorption capacity of nutrients and solutes HA present in the ECM of injured tissues helps to promote inflammation signaling in wound healing and cell proliferation and motility in wound repair [16].

HA is a signaling molecule in various etiological complications such as tumorigenesis, immune abnormalities, and inflammations. The HA-based cell signaling (Ras, c-Src, and mitogen-activated protein, MAP, kinases) is activated through the interaction of HA with RHAMM/CD44 cell surface receptors. The cell signaling property of HA is the basis for different cell functions such as cell proliferation, cell adhesion, cytoskeletal rearrangement, cell differentiation and wound healing [17]. The ability of HA in wound healing through cell signaling (*via* CD44 receptor) makes a key component in regenerative medicine [2,18]. The presence of CD44 receptors makes the internalization of HA during wound occurrence, which helps in the substrates/waste products diffusion from the injury site and helps in cell proliferation toward tight junctions formation through the interaction with the keratinocytes [19,20]. The cell signaling feature of HA with CD44 receptor also aids the internalization of fibroblasts to the wound site from the surrounding tissues, which triggers the cell signaling cascade toward the cell;s growth and motility [21]. The cell signaling property of HA with hyaluronan-mediated motility (RHAMM) receptor also aids in the wound healing process by facilitating the cellular movement through the activation of several protein kinases, which works toward tissue repair and inflammation [22,23].

Since the 1930s, HA has been majorly extracted from animal tissues like vitreous, cockscomb, umbilical cord, and others. After that, it was obtained by fermentation using pathogenic bacteria. However, both methods of HA production require extensive purification protocols to prevent toxin contamination which are expensive and may cause cross infections [24]. Recently, the possibility of producing HA from microorganisms appeared, which offered the opportunity to create a more straightforward, economical, without raw material limitations, and environmentally favorable [24]. HAs disaccharide units show the structural property in ß configuration, a very energetically stable structure with functional groups of (carboxyl, hydroxyl, acetamido, and anomeric carbon). The rigid conformation of HA;s backbone is embedded with hydrophobic -CH groups with alternate polar groups connected through hydrogen bonds (intra- and intermolecular) [6].

The comprehensive technological review on HA emphasizing the possible biosynthetic approaches, properties-based industrial applications, and the ongoing technological improvements to meet the market demands serves as the knowledge treasure for HA-based research. Hence, the present review encompassed a nutshell of HA evolution to its marketed product with the discussion of future foreseen segments for further enhanced production of HA with industrial-targeted usage with the inclusion objectives of

(i) biosynthetic approaches of HA production by microbes

(ii) insights about the utilizing fermentation strategies and DSP approaches of HA production

(iii) techno-economic aspects and industrial application scenario of HA production

(iv) prospects toward foreseeable industrial commodities as HA.

## 2. Microorganisms and biosynthesis of hyaluronic acid

HA production using microorganisms has been studied for a long time. However, some critical restrictions related to the organisms that avoided its mass production remain a big challenge. Genetic engineering, by inserting essential genes related to HA synthesis, and distinct nanoparticles have been used to increase the production of HA. In some cases, it was even possible to increase the HA production sevenfold or to increase the molecular weight of HA by partially inhibiting the glycolytic pathway and thus redirecting the carbon flux toward the production of HA [25]. However, the key constraint of producing HA of relatively uniform length has not yet been resolved. In addition, obstacles regarding the viscosity of the medium over 4 g L^−1^ of HA that limits the oxygen transfer creates an anaerobic environment that directs the carbon flow more to the production of biomass and not to HA, with the corresponding creation of contaminating by-products such as lactate [24,26]. The production of HA through different microbial routes and fermentations was summarized in (Table 1).

**Table 1.** HA production via varying microbial routes under different medium of fermentations

Microorganism	Media Composition and Production conditions	Fermentation parameters	Biomass/ HA yield	Reference
*Streptococcusequisubsp. zooepidemicus (*ATCC® 35,246™)	Glucose (45 g L^−1^), tryptone (12 g L^−1^), yeast extract (10 g L^−1^),K_2_HPO_4_ (0.2 g L^−1^), MgSO_4_·7H_2_O (0.2 g L^−1^), (NH_4_)_2_SO_4_ (0.4 g L^−1^), KH_2_PO_4_ [2 g L^−1^)	500 rpm of agitation without aeration, and pH controlled with NaCl and NaOH.	0.435 g L^−1^ with the addition of 20 mg L^−1^of Fe_3_O_4_-GA nanoparticles	7
*Lactobacillusacidophilus*PTCC1608	Lactose (20 g L^−1^], Trace elements (1.27 ml L^−1^), nitrogen sources (3 g L^−1^)	37°C without shaking	0,25 HA g L^−1^, and 1.7 g L^−1^ after response surface method (RSM) was used and a HA synthase gene was incorporated. HA was of low MW (<27 kDa).	Chahukiet al., 2019
*Bacillussubtilis*	Sucrose (20 g L-1), K_2_HPO_4_ · 3H_2_O(9.15 g L^−1^),(NH_4_)_2_SO_4_ (1 g L^−1^), Trisodium citrate·2H_2_O (g L^−1^), KH_2_PO_4_ (3 g L^−1^), casamino acids (2.5 g L^−1^), Yeast extract (10 g L^−1^), CaCl_2_ (5.5 mg L^−1^), FeCl_2_ · 6H_2_O (13.5 mg L^−1^).	Cultures were grown at 37°C, 280 rpm, and the induction process was done with xylose (0.75% wt/v) 1.5 h after incubation.	From 0.3 to around 1.0 g L^−1^ depending on the clone.	(Westbrooket al., 2018)
*Lactobacillus lactis* APJ3 (plasmid Pamj399)	Hestrin-Schramm (HS) medium (Glucose 20.0 g L^−1^, yeast extract 5.0 g L^−1^, Bacterial peptone 5.0 g L^−1^, Citric acid 1.15 g L^−1^, Sodium phosphate dibasic 2.7 g L^−1^, Magnesium sulfate 1.0 g L^−1^, pH: 5.0).	0.8 vvmwith circled silicone tubing. Media culture was circulated at 1.5 mL/min.	From 0.08 to 0.12 g L^−1^ of HA production.	(Liu andCatchmark, 2019)
*Corynebacteriumglutamicum*pXMJ19	Fermentation medium: corn syrup powder: 20 g L^−1^, glucose: 40 g L^−1^, MnSO_4_ · 7H_2_O: 10 g L^−1^, FeSO_4_ · 7H_2_O: 10 mg L^−1^, and kanamycin, 50 μg mL^−1^; (NH_4_)_2_SO_4_: 30 g L^−1^, KH_2_PO_4_: 1 g L^−1^, K_2_HPO_4_: 0.5 g L^−1^.	Fermentation medium with 2.5% v/v inoculum in 300 mL flask.After 3 h culture at 28°C, 1 mM isopropyl-β-D-thiogalactoside (IPTG) was added to induce the HA synthase expression and thus HA biosynthesis	0.49 ± 0.02 ng of HA per cell, and 5.1 ± 0.9 cells/liter (pEC-AB). 7.12 ± 0.18 ng of HA per cell, and 0.32 ± 0.05 cells/liter (pEC-AB-FtsZ).	[27]

Further research is needed to produce higher titers of HA with the same molecular weight as HA of animal sources, without impurities like microbial toxins, protein, and others [5]. The purification and further standardization of HA can receive more attention from researchers. In addition, it can be explored the use of microorganisms employing a variety of lignocellulose feedstocks to produce HA. On the other hand, the usage of microorganisms to produce HA represents a great advantage because microorganisms can proliferate, the process flow is relatively simple, unlike bovine vitreous humor or roster combs, where it is necessary to separate the complex HA-proteoglycan [24,28].

**Figure 2.**
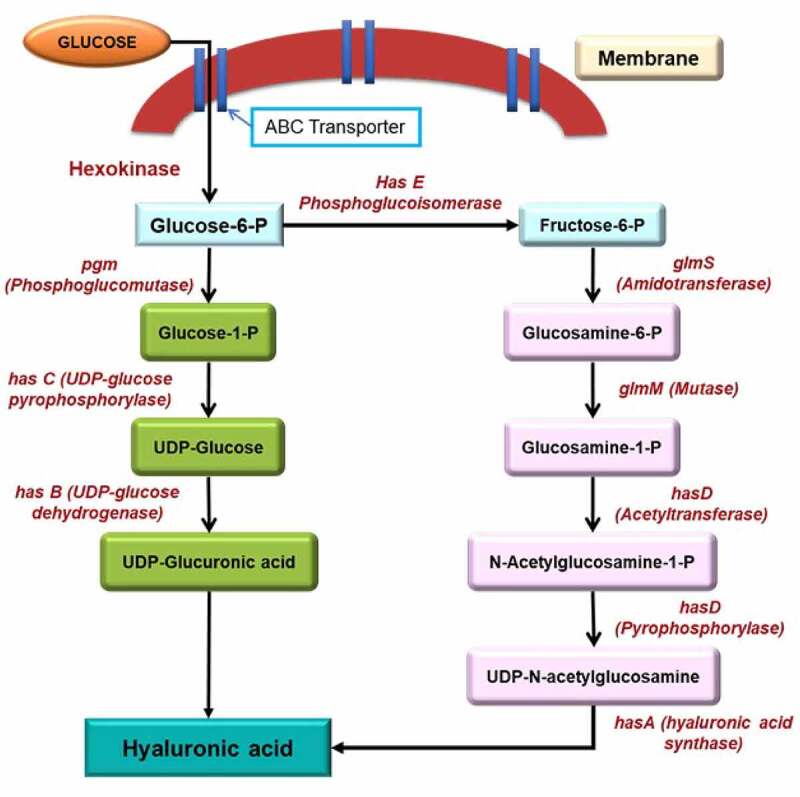
Biosynthesis of Hyaluronic acid in *S. zooepidemicus* [Modified and adapted from 78].

Usually, HA is synthesized primarily by Streptococci sp. particularly *Streptococcus equi* sub sp. *Zooepidemicus*. Streptococci is a gram-positive bacterium very diverse and heterogeneous, divided into 49 species and eight subspecies. As per the classification, the Lancefield Streptococci group is based on serological reactions of cell wall carbohydrates in different antigenic polysaccharide nature of the cell wall. In the prokaryotes, most research on the synthesis of hyaluronic acid (HA) concentrated on the *S. zooepidemicus* (Table 2). This species used two different channels to biosynthesize the precursor of hyaluronic acid [29,30]. The first step in HA biosynthesis is converting glucose to glucose-6-P (phosphate) through the hexokinase enzyme, phosphorylating glucose. The glucose-6-P is the most crucial precursor of HA synthesis. Later, this pathway parted in two different ways and formed two distinct building blocks: glucuronic acid and N-acetyl glucosamine (Figure 2). The first pathways in which glucuronic acid is involved in HA biosynthesis: started with converting glucose-6-P into glucose-1-P with the involvement of enzyme phosphoglucomutase (PGM). The enzymatic action of UDP-glucose pyrophosphorylase (*hasC*) on the glucose-1-P converted it into UDP-glucose. After that, oxidation of UDP-glucose occurs, and it forms the first precursor of hyaluronic acid, i.e., UDP-glucuronic acid, through the enzyme UDP-glucose dehydrogenase (*hasB*).

**Table 2.** HA production under different fermentation conditions by Streptococci sp

Microorganism	Fermentation Mode (capacity)	Culture medium nutrients	Fermentationparameters	Biomass/ HA yield/MW	References
*S. equi*subsp. zooepidemicus (ATCC 35246)	Batch 2 L	Marine by-products: Mussel processing wastewater (50 g L^−1^), tuna peptone (8 g L^−1^)	500 rpm No aeration	3.67 g L^−1^/ 2.46 g L^−1^/ 2.5MDa	[31]
*S. zooepidemicus*	Batch bioreactor	By-products: Sugarcane molasses and corn steep liquor	500 rpm 1 vvm pH 6.7	3.48 g L^−1^/ 3.8MDa	(Amado et al., 2017)
*S. equisubsp. zooepidemicus* (ATCC 35246)	Batch 2.5 L	Maltose (g L^−1^) increasing Carbon source consumed in CDM	600 rpm 1.3 vvm	2 g L^−1^/ 2.14 g L^−1^/ 2.1 MDa	(Chong andNelsen, 2003)
*Streptococcussp.*	Chemostatsbioreactor giving Continuousform	Chemically defined medium (CDM)	High dilution rate	HA production 25% higher than batch cultures	[32]
*S. zooepidemicus* G1 (mutant of ATCC 39920)	Batch5L with strategy plus pulsed carbohydrate added	Glucose (40 g L^−1^), polypeptone (20 g L^−1^), YE (10 g L^−1^)	10–80% DO	3.5 g L^−1^/ 2.19MDa	[33]
*S. zooepidemicus* WSH 24	Fed-batch 7 L	Sucrose (70 g L^−1^), YE (25 g L^−1^)	200 rpm 0.5 vvm	16.3 g L^−1^/ 6.6 g L^−1^/ n.d.	(Liu et al., 2009)
*S. equisubspzooepidemicus* (ATCC 39920)	Batch 10 L	Sucrose (50 g L^−1^), casein hydrolysate (10 g L^−1^)	400 rpm 2 vvm	6.5 g L^−1^/ 5.1 g L^−1^/ 3.9 MDa	[34]
*Streptococcus sp. ID9102* (KCTC 1139BP)	Batch 75 L	Glucose (4%), YE (0.75%), casein peptone (1%), Gln+Glu+ oxalic acid	400rpm 0.5 vvm	3 g L^−1^/ 6.94 g L^−1^/ 5.9 MDa	(Imet al., 2009)
*S. zooepidemicus* (ATCC 39920)	Batch 3 L	Glucose (20 g L^−1^), YE (10 g L^−1^ + acetoin and acetate	300 rpm 1 vvm	2.43 g L^−1^/ 2.15 g L^−1^/ n.d.	Wu*et al*., 2009
*S. equisubspzooepidemicus* (ATCC 35246)	Batch 2 L	Glucose (60 g L^−1^), CDM	600 rpm 1 vvm	3.5 g L^−1^/ 4.2 g L^−1^/ 3.2MDa	[35]

CDM: Chemically defined medium. NTG: N-methyl-N’-nitro-N-nitrosoguanidin. phbCAB genes: polyhydroxybutyratesynthesis genes. YE: yeast extract

The second pathway in which N-acetyl glucosamine involve in HA biosynthesis: converted the glucose-6-P into the fructose-6-P through phosphoglucoisomerase (*hasE*) enzyme. Then, amidotransferase (glmS) enzymes acted on the fructose-6-P and marked the amido group on it. Then, glutamine residue is transferred and form glucosamine-6-P. The further modification on glucosamine-6-P through enzyme mutase (glmM) changes it into glucosamine-1-phosphate. The acetylation and phosphorylation on the glucosamine-1-P through acetyltransferase and pyrophosphorylase enzymes form the second precursor of hyaluronic acid, i.e., UDP-N-acetyl glucosamine. After the formation of precursors, the enzyme hyaluronic synthase (*hasA*) proceeds to polymerize the two constituents in alternative mode and form the Hyaluronic acid polymer. For bacteria, the biosynthesis process of HA is an energy-consuming method, and numerous intermediates are also utilized for the formation of biomass, cell wall, and lactate through glycolysis [25]. The proper understanding of the microbial biosynthetic pathways helps in fermentation technologies for precise dosing of nutrients and monitoring the production parameters toward enhanced HA yields to meet the market demands of HA.

## 3. Fermentation process evolution

Different modes of fermentation (batch, repeated batch, fed batch, and continuous) have been investigated on HA production by *S. zooepidemicus* **[**36–38**]**. The intrusive effect of specific growth rate on metabolic products can be overcome by controlling the specific growth rate using continuous/fed-batch modes to obtain the higher metabolite production **[**36**]**. The usual practice of batch-based HA production **[**39,40**]** shifted toward fed-batch mode; the fermentation time was observed to be shortened with enhanced yield **[**36**]**. The utilization of continuous mode fermentation for HA production facilitates the extension of the growth cycle, waste minimization due to fermenter response time, and the reduction of MW polydispersity **[**41,42**]**. As the chain extension of HA takes place in the first half of fermentation and during the remaining fermentation, HA accumulated, a two-stage fermentation strategy was adopted for HA production with a segmented control strategy. The initial-stage fermentation was maintained at 31°C with pH 8.0 for enhancing the MW of HA, and during the accumulation stage, the temperature and pH were held at 37°C and 7.0, respectively. The proposed two-stage fermentation resulted in an optimized result with high titers of HA **[**43**]**.

**Figure 3.**
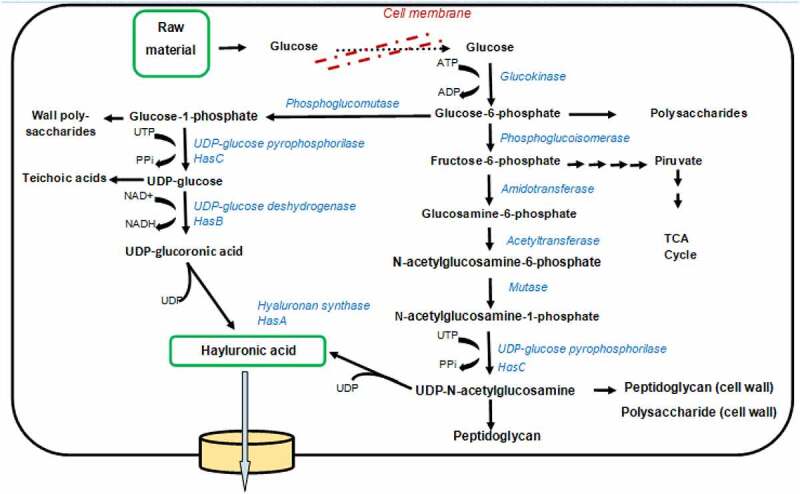
Representation of metabolic fluxes map of HA biosynthesis in *S.zooepidemicus* groups A and C(Modified and adapted from Prasad et al., 2012).

Figure 3 shows the metabolic routes for the HA production pathway in bacteria. *S. zooepidemicus* has the complete operon set containing all the genes required for HA synthesis. In wild-type *L. lactis*, the hyaluronan synthase gene is not naturally present. *L. lactis* ABC is a recombinant strain with genes that have*A,B*, and C inserted from *S. zooepidemicus. L. lactis* ABD is a recombinant strain with genes A,B, and D inserted from *S. zooepidemicus* (Prasad et al., 2012).

The industrial-scale produced HA (>1MDa), either through the animal tissue-based extraction or through the genetically modified bacterial fermentation, is suited for cosmetic and biomedical purposes [44]. The animal tissue-based extraction of HA suffers from the contamination risk with other viruses, which possess compatibility issues and further need laborious; costly DSP approaches for removal. Initially, the bacterial fermentative production of HA started with the *Streptococci C* and *A* groups but suffered from the toxins as byproducts. The next set of HA production started with the genetically modified bacteria (Gram +ve bacteria) with the insertion of HA biosynthetic pathway genes. At present, the bacterial fermentative HA is produced through the *B. subtilis*–based production system utilizing the expression constructs of *S. Equisimilis;*s *hasA* gene with the combination of overexpressed three native *B. Subtilis* precursor genes (homologous to Streptococci;s *hasB, hasC* and *hasD* genes). The HA production through genetically modified *B. subtilis* considered GRAS due to the absence of exo-endo-toxins in the production streams [45]. The HA production through the genetically modified bacterial systems is fruitful in small-scale fermenters until only 6–7 g L^−1^ yields. For further yields with large-scale fermenters results in increased media viscosity, resulting in lower mass-transfer rates with poor mixing [44]. Moreover, attaining a monodisperse-HA in bacterial-based fermentation is challenging as it depends on the culture conditions.

Due to the limitations associated with current mass production, either from animal or bacterial sources, a cell-free production system (*in vitro*) utilizing hyaluronan synthase’s (has’s) is seen as a desirable alternative. As the Class I- *has*’s are integral membrane proteins and require tedious isolation procedures and impaired functions without close association with the phospholipid layer, the peripheral-bound Class II-has;s of *P. multocida* are the viable choices toward *in vitro* production of HA on a commercial scale. The deletion of residues 704–972 (membrane domains) of Class II-has;s resulted in a ‘pmhas1-703’ (a soluble enzyme) which can make it capable of producing the HA [46]. The Class II-has;s-based in vitro (cell-free) production systems can produce ~1–2 MDa HA with low polydispersity and tuned-processability by adding HA oligomers [47]. The addition of HA oligomers circumvents the first glycosidic linkage formation (usually a rate-limiting step) by allowing the rapid acquisition of sugars to the oligomeric ends, which results in attaining a high MW-HA but fails in achieving higher yields of HA. Henceforth, various researchers attempted to clone the Class-I or -II has enzymes encoding genes in non-pathogenic host bacteria such as *E.coli* [48]. Class-I or -II *has* enzymes that act synergistically in bacterial expression systems toward HA polymer elongation [49].

With the advent of the synthetic biology approach, the construction of HA biosynthesis pathways using basic molecular components in recombinant microbes is one of the greener approaches for producing high-value HA production [50]. The biosynthesis quality of HA mainly depends on the *has*;s activity which is very crucial in constructing a HA production cell factory through synthetic biology approach. A genome-scale model (GEM) was utilized for proposing a nucleoside inosine-based HA synthesis pathway by considering the entire metabolic network of HA production by recombinant *L. lactis*, which helps in attaining a threefold enhancement in HA titer value and indicated the need for well-constructed GEMs for further improvement of HA biosynthesis [51]. A recent development in synthetic biology approach, namely rational design-based biosynthetic pathway, paves the way for uniform HA production with 6.8 g L^−1^ by implementing a two-stage induction strategy by introducing the two artificial operons (carrying *hasA gene of P. multocida) and precursor genes* (*hasB* and *hasC*) in *B. subtilis* [52]. In another synthetic biology approach, UDP-GlcNAc pathway flux enhancement results in 2 times higher HA yields (up to a specific limit of UDP-GlcNAc) by upregulating the glucose-6-phosphate isomerase (*pgi*) in *S. zooepidemicus* [53]. Carbon flux redirection is one of the approaches for inhibition of other metabolic pathways for enhanced HA yields; as a part of the approach, successful HA production results attained by reducing the expression of glycolytic pathway enzymes and facilitating the basic physiological requirements of bacteria [Zhanget al., 2016a; 98]. The combination of genetic strategies toward turning the carbon flux to HA production has been reported with the driven pathway enzymes expression, antisense RNA-mediated attenuation and knock-out pathways, additional promoter inclusions yield a 28.7 g L^−1^ HA [54]. The proposed successful synthetic biology approaches were lab-scale reports that need to be scaled up soon by developing potent HA – producing microbial strain with the synthetic biology machinery [55]. In a biorefinery context, the production of HA could be developed as an extension to the lignocellulosic biorefinery by Stereptomyces. sp through microbial conversion strategy. The fermentation technological improvements toward higher HA yields can be further accelerated by devising a perfect downstream approach (toward purified entities) for extended industrial application domains of HA.

## 4. Downstream process for HA recovery

Downstream and purification processes are essential to surpass the barrier to produce HA of high MW and purity [24]. Most reports describing HA purification are based on processes tested at the laboratory level and few at industrial scale (simulations), and in both scenarios, isopropanol is the preferred precipitating agent. The precipitation of HA is a complex process like the protein precipitation in the presence of organic solvents, where the reduction of the dielectric constant from an aqueous system to an organic solvent system favors the intermolecular interactions of the macromolecule, causing an increase of the HA molecular mass and thus its consequent precipitation [56,57]. On the other hand, at the laboratory level, the protocols imply using a detergent to liberate the capsular HA, cold ethanol to precipitate HA, and the frequent use of gel filtration chromatography to separate HA of different molecular weights [58,59].

Other procedures detail the further use of cetylpyridinium chloride to form a complex with HA, which is precipitated via centrifugation, later dissolved and treated with cold ethanol [28,60]. The methodology from 22, shows the liberation of the capsular HA with SDS (0.1%, 20 min) and its further precipitation with cold ethanol (4°C) followed by centrifugation (2000 g, 20 min).

A variation of the process involves treating the residue with varying NaCl and aqueous CPC concentrations. HA was precipitated by adding three volumes of ethanol followed by treatment with NaCl (0.01 M) and aqueous CPC (5%). The resultant mixture was then centrifuged and dissolved in NaCl (10%), dialyzed (against water), and finally, ethanol precipitated to obtain the pure HA. The recovered HA was further refrigerated (4°C, 24 h), dialyzed, and finally dried to constant weight under vacuum at 40°C [28,60]. On the other hand, the molecular weight of the HA produced is commonly evaluated with gel filtration chromatography using standards HA of various molecular weights, from 170 kDa up to 2MDa. On an industrial level, the batch fermentation showed that HA (2.5 g L^−1^ after 24 h) further centrifuged, diafiltered, activated carbon treated and finally precipitated with isopropanol [58].

Another study by 35 proposed the fed-batch fermentation coupled with simple recovery/purification (Ultrafiltration – Diafiltration, tangential flow filtration-TFF) to attain high quality and high MW HA. Downstream processing steps, i.e., Ultrafiltration – Diafiltration, TFF step, avoid the expensive chromatographic steps in the recovery of HA using 100 kDa membrane. Most of the impurities (salts, sugars, peptides) in the extracellular broth were eliminated by diafiltration mode. This step also removes the <30 kDa HA molecules harmful to cosmetic/medical applications due to pro-inflammatory properties. Finally, the TFF steps help product concentration by reducing the process volumes for further purification steps [Certminati et al., 2021; 34]. The purified HA serves as a commodity for different pharma, medical, biomaterial and cosmotic- sector with the multifold applications. The purified HA serves as a commodity for different pharma, medical, biomaterial, and cosmotic sectors with multifold applications.

## 5. Hyaluronic acid applications

HAs physicochemical/biological properties and structural and characteristic features, i.e. viscoelasticity, lubricity, biocompatibility, immunostimulation, etc, dictate the end-use of HA. The HA is a part of joint injections, osteoarthritis treatment, ocular and plastic surgeries, skin burn ingredients, and anti-aging creams [2,50,61,62]. Since 1980, HA has created intraocular prosthetic lenses [63]. The first record of injectable application occurred with the product Synvisc-produced by the company Ganzyme and approved by the US Food and Drug Administration (FDA) for viscous tissue supplementation in 2009 [63]. Another product that leveraged HA applications were Restylane, a dermal filler produced with NASHA (non-animal stabilized HA) technology that eliminates animal parts during HA extraction [63].

The development and preparation of biomaterials from HA has shown potential as a green technology, from nanofibers of this glycosaminoglycan to an agent with antitumor effects [64,& 65]. Zhu et al., 2010 [101], developed and proposed different formulations of HA as a topical or transdermal delivery agent. The authors indicate that HA obtained with low molecular weight is displayed for transdermal systems. In contrast, the increase in molecular weight increases the viscosity of the medium. It provides formulations with drugs and nutraceuticals for the topical route by forming conjugates in the form of hydrogels, nanoemulsions, microemulsions, liposomes, hydrosomes, and microneedles [66].

The different applications driven by differences in the molecular weight of hyaluronic acid were also evidenced in other studies. Molecular weights above 10 kDa are considered high and applied in ophthalmology, orthopedic, cosmetics, and tissue engineering products [67–69], while molecular weights of the order 5 kDa, considered low, are used as angiogenesis promoters, tumor inhibitors or inflammatory inhibitors [70,71].

## 6. Industrial scenario

The most important criteria for the production of HA at the industrial level is a perfect correlation between high production cost and yield of the product and the environmental risks of fermenting microorganisms. To reach high levels of purity, which is a mandatory requirement in the consumer market of HA, it is necessary to employ robust purification methods like ultrafiltration, diafiltration, adsorption with activated carbon, among others. Recently, 17 found that purification of HA aids a significant cost contribution in HA production at a large scale. It is necessary to remove the endogenous toxins Streptococci strains from the fermentation medium as they harm human health and the environment [1, 17, Ferreira et al. 2021; 62b; 83].

In this sense, HA production is market-dependent, and the primary application is related to the molecular weight of hyaluronic acid (HA). Biomedical and cosmetics applications improve HA-based products and HA-based dermal filler consumption. The HA formulated products can confer viscosity to human tissues, are easy to use with desired effectiveness. The growth of HA in healthcare is because of its demand to treat osteoarthritis, avoid cancer progress, and reverse the aging process [63,72].

The market analysis report shows that HAs global market was valued at USD 9.1 billion in 2019, which is expected to increase with a growth rate (CAGR) of 8.1% until 2027. China is a significant consumer with a record sale of 430 MT (which is more than 80% of the total world share) in 2018. Application projections show that the United States may have a USD 2.18 billion HA in the dermatological sector by 2024 [72–74].

To supply the HA for these applications, the production of HA was generally produced through tissue hydrolysis by protein removal followed by target biopolymers purification, involving mainly parameters to increase the concentration, purity and yield [61,63]. The HA animal extract was officially applied on an industrial scale in 1979 with the patent register for Balazs. This industrial approach has several unit operations like grinding, acid treatment, and extractions with organic solvents, implicating a lack of control. These severities and difficulties in controls result in some loss of size homogeneity and yield reduction. Another disadvantage is the contaminants like HA-specific binding proteins, genetic material, and disease vectors [63].

**Figure 4.**
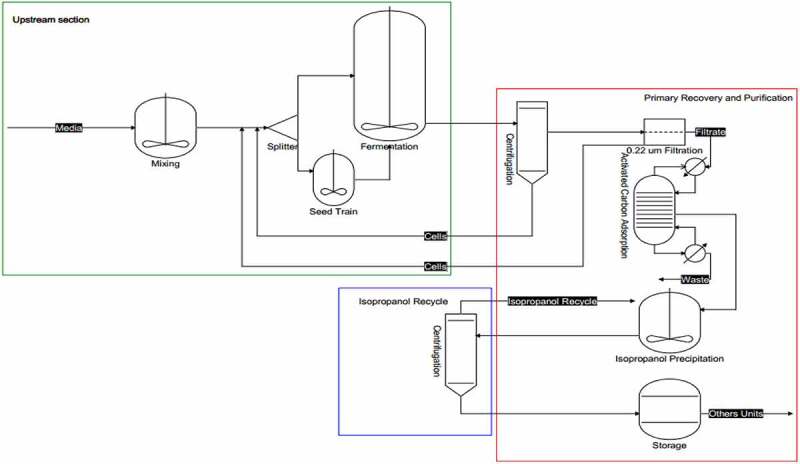
General representation of unit’s operations evolved in HA production by fermentation pathway (Modified and adapted from[77,78].

There has been an attempt to produce HA through a microbiological route employing groups *Streptococci A* and *C*, natural HA producers (Figure 4). On an industrial scale, Shiseido’s first record of HA production via the microbiological route was in 1980. The strain commonly used in the industrial plant was *S. zooepidemicus*, with the productivity of up to 7 g/L of HA under suitable fermentation conditions [44,63]. However, these strains produce numerous amounts and a variety of toxins. And more recently, fermentation strategies have used genetically modified microorganisms capable of expressing the pmHAS gene [63].

The alternative route of HA production under development since the 1990s, employing chemical routes of saccharide condensation to obtain oligosaccharides or polysaccharides, although this route suffers from difficulties with glycosylation and deprotection [61,75,76]. “The chemical synthesis of HA reported so far is the preactivation-based chemoselective glycosylation strategy employed to produce disaccharides, hexasaccharide, and decasaccharides units of HA. This methodology implies many complex steps, including using two fundamental building blocks, where the benzoyl and phthalimide groups facilitate the formation of 1,2-*trans*glycosidic bonds and a high inherent reactivity molecule as a glucuronic acid surrogate. The chemical synthesis of HA requires the control of three key factors to avoid glycosylation and deprotection that can be created because of the elongation of the sugar sequence. The strategies include performing a stereochemical control of glycosylation, introducing glucuronic acid, and finally presenting protective groups for the glucosamine nitrogen moieties [76].

Modern strategies are found in the literature involving simulations and techno-economic analysis to guide the industries for HA production (Table 3) [61,77,78]. 1 pointed out the impact of various sources and extraction methods on HA production. They concluded the potential of using animal waste and by-products as appropriate sources for extracting glycosaminoglycans (GAGs) (Table 3). 35 concluded that the downstream processes [e.g., tangential-flow and polishing filtration) is the significant cost contributing factor in HA production. Further, 17 defined that genetically modified microorganisms can make the production of HA economically viable via the microbiological route employing batch fermentation.

**Table 3.** Extraction and purification methods to obtain HA from terrestrial and marine sources

Source	Environment	ExtractionMethod	Separation/Purification method	Concentration	Reference
Eggshellmembrane	Terrestrial	Enzymatic extraction	Centrifugation	Trypsin: 44.82 mg HA/g eggshell. Papain: 39.02 mg HA/g eggshell;	(Ürgeova and Vulganova, 2016]
Eggshellmembrane	Terrestrial	Isopropanolandsodiumacetate	Silica gel and activated carbon purification	5.3 mg HA/g eggshell	(Khanmohammadi et al., 2014)
Molluskbivalve	Marine	Acetone and enzymatic extraction	Ion-exchange chromatography	4.2 mg HA/g d w of tissue	(Kanchana et al., 2013)
RoosterComb	Terrestrial	Organic solvent and sodium acetate	Centrifugation	n/d	(Kulkarni et al., 2018)
Sharkeyeballs	Marine	Alkalineprocess	Ultrafiltration, diafiltration and protein eletrodeposition	0.3 g HA/L of vitreous humor	(Murado et al., 2012)
StingrayLiver	Marine	Enzymaticextraction (Papain)	Anionexchangechromatography	6.1 mg HA/g dry weight of tissue	(Sadhasivam et al., 2013)
SwordfishEyeballs	Marine	Alkalineprocess	Ultrafiltration, diafiltration and protein eletrodeposition	0.055 g HA/L of vitreous humor	(Murado et al., 2012)

n/d: not detected

## 7. Techno-economic aspects of HA production

HA price is affected principally by its purity, which at the same time determines its different uses in various applied sectors. Application of HA in medical applications suitable for injectable applications like osteoarthritis requires HA of extremely high purity, which settles prices up to 30-fold higher than topical or dietary HA [29]. Based on reports from the US International Trade Commission and Scientific Literature, the average selling price of ‘topical’ HA is 2000 $/Kg for and for ‘injectable’ HA is 50,000 $/Kg [78].

For the microbial production of HA, wildor recombinant Streptococci sp. are being used employing various cultivation conditions. However, wild strains have the disadvantage of being zoonotic pathogens. Many research efforts have been made to improve the recombinant production of HA to minimize the side products. Three key variables have been described which influence the cost of recombinant production of HA. The method consisted in analyzing the best cost-related endogenous and recombinant production strategies of HA, followed by a sensitive analysis of production variables. It was found that production titer, recovery yield, and the bioreactor scale affected the HA production cost. It was forecasted that the recombinant production of HA needs to improve at least two-fold to compete with the endogenous production of HA [79].

Most techno-economic analyses of HA production simulated processes using wild Streptococci sp. (scenarios A, B, S1, S2, S3 and S4 in Table 4) in fermentation. However, some studies showed fermentation processes employing recombinant microorganisms (scenarios BS1 and BS2 in Table 4). Batch (scenarios S1 and S3 in Table 4) and fed batch (scenarios A, B, S2, S4, SE1, BS1 and BS2 in Table 4) fermentations were the most common systems applied in these simulations, and the volume of fermenters varied, ranging from 15.9 m^3^ (scenarios A and B), 35 m^3^ (scenarios from S1 to S4), up to 100 m^3^ (scenarios SE1, BS1 and BS2). Other scenarios considered the purity of HA produced, as in the case of scenarios B, S3, and S4, whose aim was to simulate the production of highly pure and costly injectable HA [77,78].

**Table 4.** Summary of different economic parameters for HA production using wild and recombinant microbial strains

Scenarios/economic parameters	A	B	S1	S2	S3	S4	SE1	BS1	BS2
Reference	34a		34b				17		
Technology	*Streptococci fermentation*		*Streptococci fermentation*				*Streptococci fermentation*	*B. subtillis A164Δ5*	*B. subtilis 3NA*
Total capital investment (million US$)	41.8	86.844	53.5	44.3	107	89.6	106.5	82.7	223.3
Unit production cost (US$/kg)	931	1665	1115	946	1691	1449	113	67	43
Return on investment (ROI in %)	46.12	50.64	32.6	43.5	42.5	53.1	43.6	66.4	78.3
Payback time (years)	2.17	1.97	3.07	2.30	2.35	1.88	-	-	-
Net present value (NPV) (million US$)	118.0	281.6	92.4	115.3	276.5	308.7	244.5	124.6	285.3
Product for topical use (kg/year)	20,000	17,944	20,000	20,000	19,067	19,067	1,222,357	622,912	1,426,255
Product for injectable use (kg/year)	0	2,056	0	0	871	871			
Percentual of HA diverted to injectable use	10.28%		4.36%						

The net present value (NPV) of the evaluated scenarios were all positive, emphasizing the BS2, B and SE1 scenarios. Note that the BS2 scenario, which had the highest NPV, also presented the most increased investment capital cost, reflecting the low attractiveness regarding costs of processes that employ recombinant strains in the production of HA. All processes analyzed presented high intern rates of return on investment, except scenario S1. Based on techno-economic analysis of HA production, it is recommended to produce HA in processes that employ fed-batch, wild strains – although the recombinant 3NA strain of *B. subtilis* showed a satisfactory economic profile, and whenever possible, including recycling of the reagents. Overall, the market value of HA and the means of recovering this product are the main factors that affect the economic analysis of the HA production process [63,77].

## 8. Future perspectives

HA is an anionic and non-sulfonated glycosaminoglycan widely presented in connective and epithelial tissues. HA is gaining significant market demand due to its vast cosmetic, pharmaceutical, and medical applications. It is also the main component of the group A streptococcal extracellular capsule and is believed to play a role in virulence. Further, to cater to the growing demand of HA, renewable carbon feedstocks as a carbon and nitrogen source in fermentation reactions could cut down the production cost with lower carbon footprints. Appropriate selection of lignocellulosic biomass and methods to obtain 2 G sugars followed by selecting a proper way of microbial cultivation and downstream processing to get the desired properties of HA with the optimum recovery. At the laboratory scale, the downstream process commonly uses cold ethanol (4°C) and cetylpyridinium chloride to recover the HA of higher purity. To reduce the production cost of HA on the industrial scale, isopropanol is used as solvent followed by the ultrafiltration (100 kDa) and activated carbon absorption to obtain HA of low molecular weight. Based on the techno-economic analysis, HA should be produced using fed-batch, wild strains, and with recycling of solvents in recovery.

Moreover, the market value of HA and its downstream processing to recovery are the principal elements that affect the economic analysis of HA production process. The HA production still needs to advance by creating robust, industrial host strain with the inclusion of genomic and metabolic approaches to cater to the different industrial usages of HA. Biotechnological production of HA can be further improved by employing synthetic biology strategies as an economical and sustainable alternative over traditional Streptococci fermentation and animal tissue extraction methods [24]. Firstly, the HA production still needs to be advanced by creating robust, industrial host strain with the inclusion of genomic and metabolic approaches to cater to the different industrial usages of HA. Secondly, from the viewpoint of synthetic biology, the extracellular spaces increase by changing the volume of bacterial cells in accumulated HA could be an intriguing solution [80]. Overexpression of encoding cell division inhibitor proteins (*SulA* and *MinCD*) genes in the host strains facilitate the *FtsZ* ring (Z ring) formation inhibition which in turn inhibits bacterial cell division [81] and results in a larger internal space of bacterial cells for more accumulation of HA, which boosts the sedimentation rate for easier downstream processing operations and recovery of HA [82,83]. The intrusion of synthetic biology strategies to cater to the creation of specific MW HA oligosaccharides is one of the ideal strategies for the production of industrial-targeted HA [84]. Moreover, as the efficiency of downstream operation dictates the MW and purity of HA, the traditional DSP steps need to be integrated with the physical, chemical, and membrane technologies to handle the highly viscous, non-Newtonian fermented broths and deproteinization problems in attaining a high MW, pure HA [85]. Despite mature technologies already developed for the industrial production of HA and HA-based products, the growing market, the projections of increased sales volume, and expansion of application possibilities require more efficient processes and higher HA productivity [86] [87–101].

## 9 Conclusions

HA, the hydrophilic biopolymer with the unique viscoelasticity properties, biocompatibility, water-retention, and non-immunogenic properties, has immense applications in pharma, medical, biomaterial, and cosmetic sectors. Biotechnological-based production is the viable approach for HA production and can meet the market demands if taken precise microbial selection, fermentation, and downstream properties. The biotechnological production of HA needs further research by the intrusion of synthetic biology coupled with genetic engineering approaches and technological improvements in strategic bioreactor and DSP approaches. More research has to be done to understand the mechanism action of HA in exerting biological activities and probable candidates for drug-delivery formulation and biomaterial entity with probable application domain extension of HA.
